# Time to peak postprandial glucose levels in childhood-onset diabetic patients analyzed with a continuous glucose monitoring system

**DOI:** 10.1186/1687-9856-2013-S1-P27

**Published:** 2013-10-03

**Authors:** Junichi Suzuki, Remi Kuwabara, Masako Habu, Misako Okuno, Ayako Yoshida, Tatsuhiko Urakami

**Affiliations:** 1 Department of Pediatrics and Child Health, Nihon University School of Medicine, Tokyo, Japan

## 

The risks of complications due to chronic diabetes are indicated by an increase in average blood glucose levels and the range of blood glucose level fluctuation. Postprandial hyperglycemia should be the target of glycemic control in clinical practice. There are few studies on the time to peak postprandial glucose levels in children. Therefore, we investigated the time to peak in childhood-onset diabetic patients using a continuous glucose monitor system (CGMS).

Twenty-seven patients with childhood-onset diabetes were included (male to female ratio, 9:18): 20 had type 1 diabetes, 3 had type 2 diabetes, and 4 had other types of diabetes. All patients underwent CGM on admission using the CGMS; their blood glucose levels were monitored up to 3 h after each meal. The time to peak postprandial blood glucose levels was retrospectively determined.

The CGMS recorded the postprandial glucose levels 266 times. Average peak time of postprandial blood glucose and average blood glucose excursion were 96.3 ± 56.7 min and 86.5 ± 56.7 mg/dL, respectively. The average peak time and average blood glucose excursion were as follows: type 1 diabetes, 91.2 ± 56 min and 80.0 ± 62.3 mg/dL; type 2 diabetes, 92.7 ± 42.6 min and 105.8 ± 52.2 mg/dL; other types of diabetes, 112.1 ± 50.7 min and 82.8 ± 42.7 mg/dL. The average peak time and blood glucose excursion for meals were as follows: breakfast, 108.4 ± 53.7 min and 92.6 ± 60.6 mg/dL; lunch, 93.2 ± 51.8 min and 83.4 ± 48.6 mg/dL; and dinner, 88.3 ± 54.0 min and 83.9 ± 60.0 mg/dL. There was no correlation between time to peak postprandial blood glucose and HbA1c levels.

The average time to peak postprandial blood glucose levels was approximately 90 min in both type 1 and 2 diabetes patients. The time to peak postprandial blood glucose levels reported here was delayed as compared to other studies on Japanese healthy adults with childhood-onset diabetes. The time to peak postprandial blood glucose levels in childhood-onset diabetic patients was approximately 90 min. As an indicator of glycemic control in childhood-onset diabetic patients, the CGMS was considered useful for determining the postprandial blood glucose levels in clinical practice.

